# Awareness and Preparedness of COVID-19 Outbreak Among Healthcare Workers and Other Residents of South-West Saudi Arabia: A Cross-Sectional Survey

**DOI:** 10.3389/fpubh.2020.00482

**Published:** 2020-08-18

**Authors:** Rina Tripathi, Saad S. Alqahtani, Ahmed A. Albarraq, Abdulkarim M. Meraya, Pankaj Tripathi, David Banji, Saeed Alshahrani, Waquar Ahsan, Fatimah M. Alnakhli

**Affiliations:** ^1^Department of Clinical Pharmacy, Pharmacy Practice Research Unit (PPRU), College of Pharmacy, Jazan University, Jazan, Saudi Arabia; ^2^Department of Pharmacology, College of Pharmacy, Jazan University, Jazan, Saudi Arabia; ^3^Department of Pharmaceutical Chemistry, College of Pharmacy, Jazan University, Jazan, Saudi Arabia

**Keywords:** COVID-19, coronavirus, outbreak, awareness, preparedness, healthcare, residents, questionnaire

## Abstract

**Background:** Coronavirus disease-2019 (COVID-19) was declared a “pandemic” by the World Health Organization (WHO) in early March 2020. Globally, extraordinary measures are being adopted to combat the formidable spread of the ongoing outbreak. Under such conditions, people's adherence to preventive measures is greatly affected by their awareness of the disease.

**Aim:** This study was aimed to assess the level of awareness and preparedness to fight against COVID-19 among the healthcare workers (HCWs) and other residents of the South-West Saudi Arabia.

**Methods:** A community-based, cross-sectional survey was conducted using a self-developed structured questionnaire that was randomly distributed online among HCWs and other residents (age ≥ 12 years) of South-West Saudi Arabia for feedback. The collected data were analyzed using Stata 15 statistical software.

**Results:** Among 1,000 participants, 36.7% were HCWs, 53.9% were female, and 44.1% were aged ≥ 30 years. Majority of respondents showed awareness of COVID-19 (98.7%) as a deadly, contagious, and life-threatening disease (99.6%) that is transmitted through human-to-human contact (97.7%). They were familiar with the associated symptoms and common causes of COVID-19. Health organizations were chosen as the most reliable source of information by majority of the participants (89.6%). Hand hygiene (92.7%) and social distancing (92.3%) were the most common preventive measures taken by respondents that were followed by avoiding traveling (86.9%) to an infected area or country and wearing face masks (86.5%). Significant proportions of HCWs (*P* < 0.05) and more educated participants (*P* < 0.05) showed considerable knowledge of the disease, and all respondents displayed good preparedness for the prevention and control of COVID-19. Age, gender, and area were non-significant predictors of COVID-19 awareness.

**Conclusion:** As the global threat of COVID-19 continues to emerge, it is critical to improve the awareness and preparedness of the targeted community members, especially the less educated ones. Educational interventions are urgently needed to reach the targeted residents beyond borders and further measures are warranted. The outcome of this study highlighted a growing need for the adoption of innovative local strategies to improve awareness in general population related to COVID-19 and its preventative practices in order to meet its elimination goals.

## Introduction

An ongoing outbreak of infection by Severe Acute Respiratory Syndrome-Coronavirus-2 (SARS-CoV-2), termed as COVID-19, aroused the attention of the entire world. The first infected case of coronavirus was reported on December 31, 2019, in Wuhan, China; within few weeks, infections spread across China and to other countries around the world (1). On January 30, 2020, the World Health Organization (WHO) declared the novel coronavirus outbreak a public health emergency of international concern, which was the 6th declaration of its kind in WHO history (2, 3). Surprisingly, during the first week of March 2020, devastating numbers of new cases were reported globally, and the WHO declared the COVID-19 outbreak a “pandemic” on March 11 (4, 5). The outbreak has now spread to more than 200 countries, areas, or territories beyond China (6). SARS-CoV-2 is a novel strain of the coronavirus family that has not been previously identified in humans (7). The disease spreads through person-to-person contact, and the posed potential public health threat is very high. Estimates indicated that COVID-19 could cost the world more than $10 trillion, although considerable uncertainty exists concerning the reach of the virus and the efficacy of the policy response (8).

The scientists still have limited information about COVID-19, and as a result, the complete clinical picture of COVID-19 is not fully understood yet. Based on currently available information, COVID-19 is a highly contagious disease and its primary clinical symptoms include fever, dry cough, difficulty in breathing, fatigue, myalgia and dyspnea (9–11). This coronavirus spreads primarily through respiratory droplets of >5–10 μm in diameter, discharge from the mouth or nose, when an infected person coughs or sneezes (12, 13). Reported illnesses range from very mild (including asymptomatic) to severe including illness resulting to death. However, the information so far suggested the symptoms as mild in almost 80% of the patients with lower death rates. People with co-morbidities, including diabetes and hypertension, who are treated with the drugs such as thiazolidinediones, angiotensin-converting enzyme (ACE) inhibitors, and angiotensin-II receptor blockers (ARBs) have an increased expression of angiotensin-converting enzyme-2 (ACE-2). Since, SARS-CoV-2 binds to their target cells through ACE-2, it was suggested that patients with cardiac disease, hypertension, and diabetes are at the higher risk of developing severe to fatal COVID-19 (14, 15). Moreover, elderly people (≥65 years), those and people with chronic lung disease or moderate to severe asthma, who are immunocompromised (due to cancer treatment, bone marrow or organ transplant, AIDS, and prolonged use of corticosteroids or other medications), and those people with severe obesity and chronic liver or kidney disease are at higher risk of developing the COVID-19 severe illness (16–18).

Although, no specific vaccine or treatment is approved for COVID-19, yet several treatment regimens prescribed under different conditions are reported to control the severity and mortality rates up to some extent with few adverse effects, though further evidence is needed (19). Recently, results of ongoing trials aiming at drug repurposing for the disease have been reported, and several drugs have shown encouraging activity as far as reducing the viral load or the duration of therapy is concerned. Remdesivir is one such antiviral drug, and it has reduced the duration of therapy to 11 days in comparison to 15 days in the case of patients receiving standard care only. Therefore, the USFDA has granted the emergency use authorization (EUA) to Remdesivir for the treatment of suspected or confirmed COVID-19 cases (20, 21); however, further investigations are required to collect the sufficient data (22). Favipiravir (Avigan) is another drug that has exhibited promising activity in significantly reducing the viral load in comparison to standard care in several trials (23). Apart from antiviral drugs, convalescent plasma for COVID-19 (as passive antibody therapy) has also been tested, proving to be of possible benefit in severely ill COVID-19 patients. However, it requires more clinical trials to be established for the optimal conditions of COVID-19 and as antibody therapy in this disease (24–26). Mono, and Sarilumab which are immunosuppressants and are humanized antibodies against the interleukin-6 receptor, were also tested on severely ill patients of COVID-19. They effectively improved the clinical symptoms and suppressed the worsening of acute COVID-19 patients and reduced the mortality rate (27, 28). Very recently, a corticosteroid, Dexamethasone, has been reported to be a life-saving drug that reduced the incidences of deaths by one-third among patients critically ill with COVID-19 (29) requiring oxygen support.

So far, more than 9 million confirmed cases of COVID- 19 infections have been identified globally with more than 0.46 million confirmed deaths (as on June 21, 2020). Saudi Arabia has also been seriously affected by the COVID-19 pandemic and reported its first confirmed case on March 3, 2020. The numbers are continuously increasing and reached 157,612 on June 21, 2020, with 1,267 confirmed deaths all over the kingdom (30, 31) having reproduction number from 2.87 to 4.9 (32). Before the emergence of COVID-19, Middle East Respiratory Syndrome-coronavirus (MERS-CoV) was the major concern in 2012 (33), though it was successfully controlled in Saudi Arabia. In response to the growing public health threat posed by COVID-19, the Saudi government adopted some unprecedented measures related to awareness and prevention in order to control COVID-19 transmission in the country. These measures included the closure of schools, universities, public transportation, and all public places as well as the isolation and care for infected and suspected cases (34). On March 9, 2020, government authorities announced the lockdown of the whole country and released advice for Saudi nationals and residents present inside or outside of country to stay at home and maintain social distancing. Moreover, the Saudi government decided to suspend congregational prayers across all mosques in the kingdom, including the two holy mosques in Makkah and Madinah (35).

The fight against COVID-19 continues globally, and to guarantee success, people's adherence to preventive measures is essential. It is mostly affected by their awareness and preparedness toward COVID-19. Knowledge and attitudes toward infectious diseases are often associated with the level of panic among the population, which could further complicate the measures taken to prevent the spread of the disease. As “natural hazards are inevitable; the disaster is not,” (36) to facilitate the management of the COVID-19 outbreak in Saudi Arabia, there is an urgent need to understand the public's awareness and preparedness for COVID-19 during this challenging time. The present study assessed the awareness and preparedness toward COVID-19 among South Western Saudi residents during the early rapid rise of the COVID-19 outbreak. It included HCWs (doctors, nurses, and community pharmacists) and other members of the community, including the employed, unemployed, as well as students.

## Subjects and Methods

### Setting and Population

A cross-sectional survey was conducted between March 18 and March 25—the week immediately after the announcement of lockdown in Saudi Arabia. For this study, two highly populated regions (Jazan and Aseer) of South-West Saudi Arabia and adjacent rural villages were selected. All Saudi citizens and residents, males and females of age 12 years or more (including HCWs and other community peoples), who were willing to participate in the study irrespective of COVID-19 infection status were included in the study. People who did not meet the above inclusion criteria were not eligible and were thus excluded from the study.

### Sample Size

The required sample size for this study was calculated using a Denial equation (37) where the significance level (alpha) was set to 0.05 and power (1-β) was set to 0.80. It resulted in a required final sample size of 384 individuals. Therefore, to minimize the errors, the sample size taken for this study was 1,000.

### Outcome Measures

The present study examined the level of awareness and preparedness toward prevention of COVID-19 using area, gender, age, education level, and occupation as explanatory variables among the residents (HCWs and other community peoples) of South-West Saudi Arabia.

### Study Tool

Since this is a novel coronavirus with no such study having been conducted before, a standardized (structured, pre-coded, and validated) questionnaire was developed for this study by our co-authors, and it is based on frequently asked questions (FAQ) found on Centers for Disease Control (CDC) and WHO official websites (38, 39). The questions were multiple choice and sought to gain insight into the respondent's awareness and preparedness toward COVID-19. A pilot survey of 10 individuals was undertaken first to ensure that the questions elicited appropriate response and there were no problems with the entry of answers into the database. Since, it was not feasible to conduct a community-based national sampling survey during this critical period; we decided to collect the data online through a Google survey. The self-reported questionnaire is divided into three sections. The first part is designed to obtain background information, including demographic characteristics (nationality, age, gender, level of educational, and occupation). The second part of the survey consists of questions that address awareness concerning COVID-19 (reliable source of information, symptoms, mode of transmission, incubation period, complications, high-risk population, treatment, and preventive measures). The third part of the survey consists of questions that address the preparedness to fight against COVID-19. The questionnaire is designed in English, being subsequently translated into Arabic for the convenience and ease of understanding of the participants, and it was pre-tested to ensure that it maintained its original meaning.

### Data Collection and Analysis

Data were collected using a random sampling method and analyzed using the statistical software Stata 15. For categorical variables, data were presented as frequencies and percentages. A chi-squared (χ^2^) test was used to examine the association between each item in awareness and explanatory variable in the bivariate analysis. Multivariable logistic regression was computed using each item in awareness and preparedness as an outcome separately to examine the relationships in the adjusted analysis. Differences were considered to be statistically significant at *P* ≤ 0.05.

### Ethical Approval

The study protocol and procedures of informed consent were granted ethical approval by the “Institutional Research Review and Ethics Committee (IRREC), College of Pharmacy, Jazan University” before the formal survey was conducted. Since this study was conducted during the lockdown period, a Google survey was prepared with an online informed consent form on the first page. Participants are informed about the contents of the questionnaire, and they have to answer a yes/no question to confirm their willingness to participate voluntarily. In case of minors (participants below 16 years of age), they are asked to show the form to their parents/guardians before selecting their answer. The patients/participants or their legal guardians have to provide their written informed consent to participate in this study. After an affirmative response of the question, the participant is directed to complete the self-report questionnaire. All responses are anonymous.

## Results

### Demographic Characteristics

Respondents' demographic descriptions are summarized in Table 1. A total of 1,000 participants completed the survey questionnaire, the split being 46.1% male and 53.9% female. The majority of participants are from Jazan region (74.8%) compared to 25.2% from Aseer province. More than half (55.9%) of the participants are of <30 years of age, and 44.1% are aged ≥ 30 years. Around 79.5% respondents are university graduates holding a bachelor's degree or higher, whereas 20.5% of participants possess educational qualifications of secondary school or lower (non-graduates). HCWs make up 36.7% of participants, and 63.3% of participants are classified as other.

**Table 1:** Socio-demographic characteristics of participants.

**Variable**		**Count (*n*)** ***n* = 1,000**	**Percentage** **(%)**
Region	Jazan	748	74.8
	Asser	252	25.2
Gender	Male	461	46.1
	Female	539	53.9
Age groups	<30 years	559	55.9
	≥30 years	441	44.1
Education	Middle school or less	26	2.6
	High School	179	17.9
	Bachelor Degree	634	63.4
	Master/Ph.D./above	161	16.1
Occupation	Doctor	76	7.6
	Nurse	51	5.1
	Pharmacist	240	24.0
	Other Employed	238	23.8
	Unemployed	123	12.3
	Students	272	27.2

*n, Number of participants*.

### Knowledge of COVID-19 Disease and Personal Protection Measures

Table 2 displays respondents' knowledge about COVID-19, reliable sources of information, modes of transmission, symptoms of infection and complications, its perceived threat, and high-risk population. Respondents were allowed to choose more than one option from the choices given according to their understanding and conscience. The results indicated that majority of respondents had heard of and were aware of COVID-19 disease. Most of the participants (97.7%) correctly identified human-to-human transmission (contaminated person with virus) as the primary mode of transmission. Furthermore, fever, cough, and difficulty in breathing were stated as the most common COVID-19 symptoms by 89.8, 83.9, and 90.9% of respondents, respectively. The frequently reported complications of COVID-19 were pneumonia (79.4%), kidney failure (22.8%), and death (54.9%) by the respondents.

**Table 2:** Awareness about COVID-19, its symptoms, transmission, and complications.

**Variable**		**Count (*n*)** ***n* = 1,000**	**Percentage** **(%)**
1. Heard of COVID 19	Yes	987	98.7
	No	13	1.3
2. COVID 19 is contagious life threatening disease	Yes No	996 4	99.6 0.4
3. Incubation period	2–14 days	957	95.7
	3 weeks	63	6.3
	≥1 month	11	1.1
	Don't know	32	3.2
4. Reliable source of information^*^	Health organization	896	89.6
	Healthcare professionals	579	57.9
	Social media	155	15.5
	Television/you tube	124	12.4
	Newspaper/Poster	30	3.0
	Family/friends	27	2.7
	Don't know	9	0.9
5. Mode of transmission^*^	Human-to-human transmission	977	97.7
	Animals contact	229	22.9
	Sea food and live animal	128	12.8
	Fast food	93	9.3
	Domestic animal	45	4.5
	Don't know	24	2.4
6. Symptoms of COVID 19^*^	Difficulty in breathing	909	90.9
	High temperature/Fever	898	89.8
	Cough	839	83.9
	Sore throat	542	54.2
	Tiredness	531	53.1
	Pain in the muscles	343	34.3
	Runny nose	217	21.7
	Common cold	203	20.3
	Nausea/Vomiting	179	17.9
	Don't know	20	2.0
7. Complications^*^	Pneumonia	794	79.4
	Kidney failure	228	22.8
	Sepsis and septic shock	76	7.6
	Visual/Memory loss	18	1.8
	Death	549	54.9
	Don't know	123	12.3

* *Multiple answers were possible; n, Number of participants*.

Participants' knowledge of personal protection against COVID-19 is summarized in Table 3. The majority of respondents (76.4%) believe that there is no treatment available for COVID-19 to date, 47.1% report supportive care, and 45.8% state personal safety as the only treatment option. The most common personal protection practices adopted by participants are washing hands (92.7%), social distancing (92.3%), using a face mask (86.5%), and avoiding travel to infected areas or countries (86.9%). However, importantly, 63.8% participants believe in avoiding raw and under-cooked animal products, 16.2% choose to avoid purchasing products made in China, and 1.7% have knowledge of proper prevention methods. Approximately, half of the respondents (42.4%) report that they seek more information on COVID-19.

**Table 3:** Awareness about personal protection and preparedness against COVID-19.

**Variables**	**Count (*n*)**	**Percentage**
	**(*n* = 1,000)**	**(%)**
**What is the current situation in Saudi Arabia regarding COVID-19**		
Many cases have been reported till date	967	96.7%
No case have been reported till date	8	0.8%
Don't know	25	2.5%
Population at high risk^*^		
Elderly age ≥ 65 years	745	74.5
People with comorbid conditions	686	68.6
Health care workers or other who cares infected patient	560	56.0
Live animal market workers	240	24.0
Personal protection measures^*^		
Social distancing (avoid personal contact)	923	92.3
Hand hygiene (washing hands more often)	927	92.7
Use face mask (cover nose)	865	86.5
Avoid travel to infected area or country	869	86.9
Avoid visiting wet markets, raw and under cooked animal products	638	63.8
Avoid sea food and live animals etc.	200	20.0
Use different chopping board and knives for raw meat and other food	183	18.3
Avoid purchasing things made in china	162	16.2
Avoid vegetarian food	32	3.2
Don't know	17	1.7
Treatment available for COVID-19^*^		
No treatment/vaccine till date	764	76.4
Supportive treatment	471	47.1
Just keep yourself safe	458	45.8
Vaccination	17	1.7
Don't know	86	8.6
Preparedness to fight against COVID-19^*^		
Avoiding mass gathering and traveling to suspected area	951	95.1
Using hand sanitizer, face mask, home cleaning materials recent days	827	82.7
Spending 20 s thoroughly for washing hands now a days	768	76.8
Maintaining food hygiene	611	61.1
Stored food items and basic required things in home as its lockdown	447	44.7
Ready to visit hospital immediately if needed	555	55.5
Do you need more information about prevention of COVID 19?		
Yes	424	42.4

* *Multiple answers were possible; n, Number of participants*.

### Preparedness to Fight Against COVID-19

Results of participants' preparedness against COVID-19 are summarized in Table 3. Over one-third of participants are well-prepared and adopt various methods for the current situation. The majority of participants stat that they avoid crowded places, mass gatherings, or traveling to suspected areas (95.1%), and 82.7% wear face masks when going outside and have increased the use of hand sanitizers and home cleaning materials. Many of them (76.8%) now spend 20 seconds washing their hands using soap multiple times a day. However, it could be assumed from the survey that a considerable percentage of the participants do not find the protective measures necessary, visit crowded places, and do not wear face masks when leaving home.

**Figure 1:**
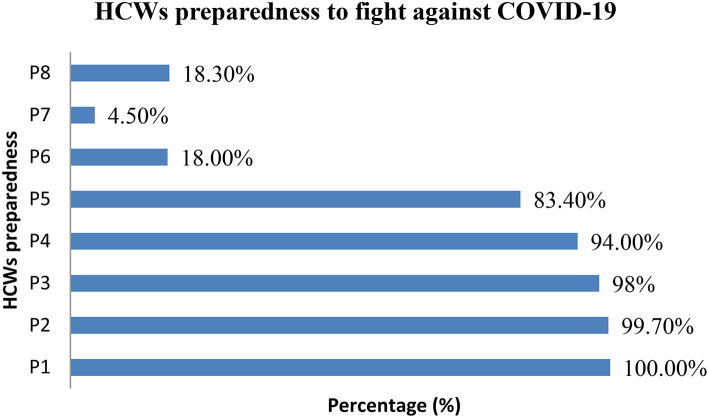
HCWs preparedness to fight against COVID 19. P1, Check adequate supplies of goggles, masks, and gowns on hand for emergencies. P2, Links to or contact External Resource Centers for COVID-19 (Coronavirus) (CDC, WHO etc.). P3, Check patient care equipment, including portable ventilators. P4, Recommendations for infection control to help biomedical and clinical engineers. P5, Check alternative suppliers of certain personal protective equipment. P6, Prepared the list to supply chain professionals. P7, Do not need any preparation. P8, I don't know.

On the other hand, HCWs also reported their preparedness on different areas to fight against COVID-19 (Figure 1). All 367 (100%) HCWs who participated in this study say that they checked adequate supplies of goggles, masks, and gowns on hand for emergencies, 99.7% say they prepared links or are in contact with External Resource Centers for COVID-19 such as the CDC or WHO, 98% evaluated the patient care equipment, including portable ventilators (preparation and patient handling checklists), and 83.4% checked and prepared alternative suppliers list of certain personal protective equipment etc. Surprisingly, 18.3% of the respondents are unaware of any preparation, and 4.5% do not find it necessary.

### Bivariate Analysis

The comparison between educational groups and occupational groups (HCWs vs. other residents) demonstrated significant differences in the level of knowledge and preventive measures for COVID-19 disease (Tables 4, 5). The survey shows educated participants (bachelors or more) and HCWs were more aware about COVID-19 symptoms (*P* ≤ 0.001), incubation period (*P* ≤ 0.001), complications (*P* ≤ 0.001), high-risk populations (*P* ≤ 0.01), and available treatment (*P* ≤ 0.05) compared to less-educated (≤high school) ones and other residents (non HCWs). Jazan area participants heard about (*P* ≤ 0.002), and showed more awareness regarding COVID-19 symptoms (fever: *P* ≤ 0.001), and available treatment (supportive care: *P* ≤ 0.001) as compared to the Aseer region. There were no significant differences found in knowledge level between gender (male vs. female) and age groups.

**Table 4:** Awareness of COVID-19 stratified by occupation groups among the study participants (*n* = 1,000).

**Knowledge**		**Occupation groups**		***P*-value**
	**Total**	**HCWs**	**Other residents**	
	***n*= 1,000**	***n* = 367**	***n* = 633**	
Incubation period : 2–14 days	957	362 (98.64%)	595 (94%)	0.001
**Symptoms**				
Difficulty in breathing	909	349 (95.1%)	560 (88.47%)	0.001
High temperature/Fever	898	349 (95.1%)	549 (86.73%)	0.001
Cough	839	327 (89.1%)	512 (80.88%)	0.001
Sore throat	542	215 (58.88%)	327 (51.66%)	0.034
Tiredness	531	225 (61.31%)	306 (48.34%)	0.001
Pain in the muscles	343	166 (45.23%)	177 (27.96%)	0.001
Runny nose	217	106 (28.88%)	111 (17.54%)	0.001
**Complications**				
Pneumonia	794	327 (89.1%)	467 (73.78%)	0.001
Kidney failure	228	108 (29.43%)	120 (18.96%)	0.001
Sepsis and septic shock	76	41 (11.17%)	35 (5.53%)	0.001
**Population at high risk**				
Elderly age ≥ 65 years	745	292 (79.56%)	453 (71.56%)	0.005
People with comorbid conditions	560	238 (64.85%)	322 (50.87%)	0.001
Health care workers	686	277 (75.48%)	409 (64.61%)	0.000
**Personal protection measures**				
Social distancing	923	347 (94.55%)	576 (91%)	0.042
Use face mask	867	331 (90.19%)	534 (84.36%)	0.009
Avoid purchasing things made in china	162	41 (11.17%)	121 (19.12%)	0.001
**Treatment available for COVID 19**				
No treatment till date	764	299 (81.47%)	465 (73.46%)	0.004
Supportive treatment	471	229 (62.4%)	242 (38.23%)	0.001

*P < 0.05 is considered statistically significant (Chi square test), n, Number of participants; HCWs, health care workers (including doctors, nurses, and pharmacist), Other residents including employed, unemployed, and students*.

**Table 5:** Awareness of COVID-19 stratified by educational groups among the study participants (*n* = 1,000).

**Knowledge**	**Total *N* = 1,000**	**Educational groups**			***P*-value**
		**≤High school *n* = 205**	**Bachelor degree *n* = 634**	**Master/Ph.D. or above *n* = 161**	
Incubation period : 2–14 days	957	183 (89.27%)	616 (97.16%)	158 (98.14%)	0.001
**Symptoms**					
Difficulty in breathing	909	172 (83.9%)	582 (91.8%)	155 (96.27%)	0.001
High temperature/Fever	898	170 (82.93%)	571 (90.06%)	157 (97.52%)	0.001
Cough	839	158 (77.07%)	533 (84.07%)	148 (91.93%)	0.001
Sore throat	542	101 (49.27%)	325 (51.26%)	116 (72.05%)	0.001
Tiredness	531	83 (40.49%)	351 (55.36%)	97 (60.25%)	0.001
**Complications**					
Pneumonia	794	136 (66.34%)	515 (81.23%)	143 (88.82%)	0.001
Kidney failure	228	32 (15.61%)	136 (21.45%)	60 (37.27%)	0.001
Sepsis and septic shock	76	6 (2.93%)	49 (7.73%)	21 (13.04%)	0.001
**Population at high risk**					
Elderly age ≥ 65 years	745	141 (68.78%)	471 (74.29%)	133 (82.61%)	0.010
People with comorbid conditions	686	111 (54.15%)	444 (70.03%)	131 (81.37%)	0.001
Health care workers	560	94 (45.85%)	364 (57.41%)	102 (63.35%)	0.002
**Personal protection measures**					
Social distancing	923	178 (86.83%)	595 (93.85%)	150 (93.17%)	0.004
Avoid travel to infected area or country	869	165 (80.49%)	564 (88.96%)	140 (86.96%)	0.008
Avoid things made in china	162	51 (24.88%)	102 (16.09%)	9 (5.59%)	0.001
**Treatment available for COVID 19**					
No treatment till date	764	138 (67.32%)	487 (76.81%)	139 (86.34%)	0.001
Supportive treatment	471	61 (30.24%)	317 (50%)	92 (55.14%)	0.001

*P < 0.05 is considered statistically significant (Chi square test), n, Number of participants; HCWs, health care workers (including doctors, nurses, and pharmacist), other residents: including employed, unemployed, and students*.

Significant differences were observed in awareness about protective measures between educational groups and occupational groups (Tables 4, 5). The survey shows that the educated participants (Bachelors or more) and HCWs consider the use of face masks, frequent washing of hands, social distancing, and avoid traveling to an infected area or country as preventive measures, more so than their counter group (*P* < 0.05). However, gender, age, and area comparisons on these measures were non-significant. Moreover, the survey exhibited no significant differences regarding preparedness to fight against COVID-19 level between areas, age, gender, and educational and occupational groups.

### Multivariable Logistic Regressions

It was found that HCWs were more likely to be aware of COVID-19 symptoms (fever: OR = 2.15, *P* = 0.008; cough: OR = 1.66, *P* = 0.018 etc.), complications (pneumonia: OR = 2.37, *P* = 0.001; kidney failure: OR = 1.54, *P* = 0.013 etc.), populations at high risk, available treatment, and preventive measures compared to the other community members who were non-HCWs. On the other hand, less-educated participants (≤secondary schooling) were more likely to have knowledge about COVID-19 symptoms (fever: OR = 4.24, *P* = 0.014; breathing difficulty: OR = 2.94, *P* = 0.043 etc.), high-risk population (OR = 3.29, *P* = 0.001), complications, and preventive measures (social distancing: OR = 2.08, *P* = 0.008; avoid traveling to infected area or country: OR = 2.01, *P* = 0.002 etc.) compared to the higher-educated participants, as shown in Tables 6, 7. Tables displayed outcomes with statistically significant association only with explanatory variable. Area (Jazan vs. Aseer), gender (male vs. female), and age group (age <30 years vs. ≥30 years) were not associated significantly with COVID-19 knowledge. Surprisingly, no difference was reported for preparedness to fight against COVID-19 among participants.

**Table 6:** Multivariable logistic regression on factors significantly associated with awareness toward COVID-19.

**Explanatory variable**		**Odds ratio (CI 95 %)**	***P-*value**	**Odds ratio (CI 95 %)**	***P-*value**	**Odds ratio (CI 95 %)**	***P-*value**
**Outcome: Symptoms**		**High temperature (Fever)**		**Cough**		**Difficulty in breathing**	
Area	Asser	[1] Reference		NS	–	NS	–
	Jazan	1.61(1.02–2.53)	0.040	–	–	–	–
Education level	≤ High school	[1] Reference		[1] Reference		[1] Reference	
	Bachelor degree	1.44(0.90–2.31)	0.126	1.33(0.89–2.01)	0.164	1.88(1.15–3.06)	0.011
	Master/PhD/above	4.24(1.34–13.43)	0.014	2.33(1.08–5.00)	0.030	2.94(1.03–8.40)	0.043
Occupation	Other residents	[1] Reference		[1] Reference		[1] Reference	
	HCWs	2.15(1.22–3.79)	0.008	1.66(1.09–2.53)	0.018	1.92(1.08–3.43)	0.026
**Outcome: Complication**		**Pneumonia**		**Kidney Failure**		**Sepsis**	
Education level	≤High school	[1] Reference		[1] Reference		[1] Reference	
	Bachelor degree	1.72(1.19–2.48)	0.004	1.31(0.85–2.04)	0.224	2.34(0.97–5.68)	0.060
	Master/Ph.D./above	2.68(1.36–5.32)	0.004	2.41(1.31–4.44)	0.005	3.42(0.03–1.15)	0.027
Occupation	Other residents	[1] Reference		[1] Reference		[1] Reference	
	HCWs	2.37(1.58–3.57)	0.001	1.54(1.09–2.16)	0.013	1.68(1.00–2.82)	0.049
**Outcome: Treatment available**		**Supportive care only**		**Just keep safe**		**No treatment**	
Education level	≤High school	[1] Reference		[1] Reference		[1] Reference	
	Bachelor degree	1.73(1.22–2.47)	0.002	0.79(0.57–1.11)	0.184	1.54(1.07–2.22)	0.019
	Master/Ph.D./above	1.89(1.10–3.23)	0.020	0.55(0.32–0.93)	0.027	3.27(1.67–6.41)	0.001
Occupation	Other residents	[1] Reference		–	–	–	–
	HCWs	2.26(1.68–3.03)	0.001	NS	–	NS	–
**Outcome: High risk population**		**Patients with comorbidities**		**Health care professional**			
Education level	≤High school	[1] Reference		NS	–		
	Bachelor degree	1.81(1.29–2.54)	0.001	–	–		
	Master/Ph.D./above	3.29(1.83–5.90)	0.001	–	–		
Occupation	Other residents	[1] Reference		[1] Reference			
	HCWs	1.36(0.99–1.87)	0.001	1.61(1.20–2.16)	0.001		

*P < 0.05 is considered statistically significant, HCWs, health care workers (including doctors, nurses, and pharmacist), other residents: including employed, unemployed, and students; NS, Non-significant result*.

**Table 7:** Multivariable logistic regression analysis on factors significantly associated with preventive measures toward COVID-19.

**Explanatory variable**		**Odds ratio (CI 95%)**	***P-*value**
**Outcome: Preventive measures**			
**Wash hand frequently**			
Education level	≤High school	[1] Reference	
	Bachelor degree	1.75(1.15–2.66)	0.009
	Master/Ph.D./above	4.15(1.67–10.33)	0.002
Occupation	Other residents	[1] Reference	
	HCWs	1.90(1.17–3.09)	0.010
**Use face mask**			
Occupation	Other residents	[1] Reference	
	HCWs	1.55(0.99–2.43)	0.055
**Avoid personal contact with infected peoples**			
Education level	≤High school	[1] Reference	
	Bachelor degree	2.08(1.21–3.59)	0.008
	Master/Ph.D./above	1.41(0.57–3.50)	0.457
**Avoid travel to infected area or country**			
Education level	≤High school	[1] Reference	
	Bachelor degree	2.01(1.29–3.16)	0.002
	Master/Ph.D./above	1.43(0.69–2.96)	0.334
**Avoid purchasing things made in China**			
Education level	≤High school		
	Bachelor degree	0.64(0.43–0.95)	0.028
	Master/Ph.D./above	0.26(0.11–0.62)	0.002

*P < 0.05 is considered statistically significant, HCWs, health care workers (including doctors, nurses, and pharmacist), other residents: including employed, unemployed, and students*.

## Discussion

As the outbreak of COVID-19 is expanding exponentially, spreading beyond borders and spreading across continents, it has been classified as a “pandemic.” It created havoc and dismay among all nations. This new viral infection is successful in inducing restlessness, confusion, and fear among the people. The uniqueness of this infection is that it shows little or no symptoms in the beginning, and many do not even know they are infected. It does not induce any severe change or indication in the infected person so that he can seek medical attention at an early stage. By the time infected persons realize that they are infected, they might have spread the disease to a large number of people without their knowledge and any ulterior motives. Therefore, the first and foremost strategy to win the battle over COVID-19 shall be stopping the spread of disease effectively among the people. Hence, the main focus of this research was to assess the awareness of people, particularly among HCWs as well as other residents, about the disease, how they prepared themselves to fight against it, and whether they are participating in the eradication of the infection or not. We are aware that COVID-19 had taken the nation by surprise when they were least prepared to face the pandemic. To the best of our knowledge, this is the first study of its kind, conducted in Saudi Arabia that is assessing the awareness and preparedness toward COVID-19 among HCWs and other residents.

Our survey of HCWs and other residents of the study region was well-received. People of different educational backgrounds and employments participated in the survey. The majority of them are graduates, followed by people who had education up to high school. Similarly, among different employment backgrounds, HCWs make up more than one-third of the sample size. In the first place, HCWs and graduates should be aware of the disease profile, so that they can quickly spread the message among their family members, their neighbors, and all those who are within their contact. Analysis of the study results showed that both HCWs and the graduates possess adequate knowledge about the infection. It was a significant finding of our study that they can not only protect themselves against the disease but also help others to stay away from the infection by creating awareness for it. As the results suggested, health organizations (89.6%) and healthcare professionals (57.9%) are able to communicate effectively to the participants in convincing and making them understand the patterns and phases of the infection. This study also revealed that some people showed little trust in social media and other sources of communications such as television, newspaper, posters, etc. They were not convinced or accepting of the facts disseminated to them initially. It is probably for this reason that few people showed reluctance in following the guidelines given through these channels and kept ignoring them. This lack of acceptance might have accelerated the spread of this disease among the public.

Our study revealed that HCWs and people with a higher educational background (graduation or more) were more aware of the symptoms and the complications of COVID-19. It is spread via human-to-human transmission through droplet, feco-oral, and direct contact and has an incubation period of 2–14 days (13). The majority of the participants (97.7%) mentioned human-to-human contact as the primary cause of COVID-19 transmission. They were aware that the infection is related to the respiratory system, and there could be some difficulties in breathing with high temperatures accompanied by dry cough. Furthermore, it might lead to pneumonia, organ failure, and death. Indeed, COVID-19 induces these symptoms after the log period (40), although in some cases. Also, HCWs keenly follow the situation in the regions and the countries regarding the number of cases of infected and fresh cases reported daily. It perhaps helps them in getting prepared physically to manage the situation by acquiring the important things that are required in combating the disease, and it might also help them to get prepared mentally. They were aware of the social distancing, hand hygiene, using face masks, and avoiding traveling. These are the desired activities, which are expected to be practiced strictly in order to stop the spread of the disease (41–44). Our study revealed that HCWs and educated residents were following it meticulously. It was also known to them that no specific and effective treatment is available for COVID-19 to date, and whatever therapy is available at the designated centers is non-specific and treats only symptoms. They are sufficient enough to relieve the symptoms of the infection, to overcome difficulties in breathing, and to boost the immunity of the individuals. A similar level of awareness was reported in recent studies in China (10) and the UAE (45). This may be attributed to continuous practice of raising awareness about COVID-19 in communities about health issues by healthcare organizations and Saudi health extension workers, which has been effectively implemented in recent days (46).

Previously, MERS-CoV was a major global concern after it was first identified in 2012 in Saudi Arabia (33). Many awareness studies reported different levels of knowledge about MERS disease among Saudi HCWs and residents after the MERS outbreak (47–49). Present findings showed that the awareness regarding COVID-19 disease was higher compared to MERS. This can be ascribed to the global reach of COVID-19, as it is more serious than MERS owing to its high rate of transmission, alarming number of cases, and the continued global death count.

As far as preparedness to fight against COVID-19 is concerned, our study showed that all the participants were aware of avoiding mass gathering, avoiding traveling to suspected areas, the use of face masks and hand sanitizers, and maintaining proper food hygiene. During the lockdown period, the majority of the people who participated in our study stockpiled sufficient food items, and the frequency of going out to buy groceries and other food items can thus be avoided. According to them, a large number of people at supermarkets do not practice appropriate social distancing, and chances of contracting the infection might increase. This is genuinely desirable and precautionary in a situation like COVID-19, as coming closer to or violating social distancing is risky. Perhaps this preparedness is a reflection of steps taken by government authorities, as Saudi Arabia can control the spread of COVID-19 in South-West region. When the whole world is struggling to control COVID-19 spreading, Saudi Arabia has reported 1,155 positive cases (as of June 21, 2020) in Jazan and Aseer region (313 and 842, respectively) among 157,612 positive cases the entire country (50).

Also, our study confirmed that nearly half of the participants were ready to visit the hospital immediately if needed. The WHO recommends that identification of the infected individual is the first and essential step required in combating COVID-19. It also advises nations to allow citizens to get tested and put them in quarantine if they are infected. It is a significant step, as nearly 50% of the people are aware of the importance of testing in suspected cases but the remaining 50% of the participants are not. Doubts or fears about quarantine can make the public hide behind closed doors. This behavior of theirs could be dangerous, as it not only puts them in a difficult situation, but is risks their entire family and neighbors. Surprisingly, nearly 42.4% of the participants have asked for more information about COVID-19 so that they can take sufficient precautions and prepare themselves to avoid contracting the disease. These are the participants who had fewer opportunities to access healthcare services. They indeed need more information on COVID-19 to stay away from the deadly disease. This is the substantial finding of our study: nearly half of the participants did not have detailed information or a desire to gain more knowledge about the disease. The focus of the administrators should be on this category of people—the common man—so that they too can prepare themselves to fight the disease. Overall, the reported preparedness could be because the healthcare authorities have already initiated awareness and preparedness activities beyond their own borders. Every country around the world is being encouraged to draft a preparedness plan as per the WHO's global guidelines: “The ‘COVID-19' Strategic Preparedness and Response Plan” (SPRP). The SPRP outlines the public health measures that are needed to be taken to support countries to prepare for and respond to COVID-19 (51, 52).

It was observed that the educational background plays a significant role in understanding the infection quickly. This survey showed that HCWs and people with higher education have a better understanding of the disease than their counterparts. Even though all the groups showed almost identical knowledge about the primary information of the disease, in some areas, such as disease complications, high-risk populations, personal protection measures, and treatment availability, a clear distinction exists. For example, only 68.78% of the less educated showed awareness of the high risk of contracting the infection of older people.

The WHO have initiated several online training sessions and materials on COVID-19 in various languages to strengthen preventive strategies, including raising awareness and training HCWs in preparedness activities (53). In several instances, misunderstandings among HCWs have delayed controlling efforts to provide necessary treatment (44), which led to the rapid spread of infection in hospitals (33, 49) and putting patients' lives at risk. The present study also analyzed the preparedness of HCWs to fight against COVID-19 and found all participated HCWs were well prepared and ready for the current outbreak.

All participating HCWs report that they have adequate supplies of personal protective equipment's (PPEs), such as goggles, masks, and gowns, to manage emergencies, 99.72% of HCWs depend on an external resource center like CDC and WHO for the required emergency materials, and 98% HCWs say that they have already checked their hospitals equipped with patient care equipment, including portable ventilators. Surprisingly, few respondents (18.26%) say they were unaware of any preparation, and very few (4.36%) say that there is no need for any preparation. In general, our study indicated that the HCWs have well equipped themselves to fight against COVID-19. Although, hospitals and HCWs are fully geared up to face the pandemic situation, the best national option available is to spread awareness in order to stop the spread of disease. We have no other way but to educate our fellow citizens to not indulge in any activities that could lead them being a part of the problem. Instead, they should be encouraged to be the part of the solution.

The WHO has published guidance for public health and social measures at the workplace within the context of COVID-19. This included the standards for all workplaces and specific criteria for workplaces and jobs at medium risk and high risk. The guidance suggested to adapt the essential preventive measures for all workplaces, including practicing hand hygiene, respiratory hygiene, physical distancing (avoid direct physical contact by hugging, touching, or shaking hands), reducing and otherwise managing work-related travel, regular environmental cleaning and disinfection, risk communication, training and education, and management of people with COVID-19 or their contacts. In addition, specific measures for workplaces and jobs at medium risk included frequent cleaning and disinfection of objects and surfaces that are touched regularly (fomites). In such places where physical distancing of 1 meter cannot be maintained for a particular activity, all mitigating actions possible should be taken to reduce the risk of transmission between workers, clients or customers, contractors, and visitors, and these include staggered activities, minimizing face-to-face and skin-to-skin contact, ensuring workers work side-by-side or facing away from each other rather than face-to-face, and assigning staff to the same shift teams to limit social interaction. Along with that, such workplaces must be well-ventilated with a natural air of artificial ventilation without re-circulation of air for high-risk work activities and jobs. The WHO have advised that we find possibilities to suspend operations or adhere to the hygiene measures before and after contact with or suspicion of COVID-19. In such cases, workers must comply with the use of medical masks, disposable gowns, gloves, and eye protection for workers and use of protective equipment when in contact with COVID-19 patients, their respiratory secretions, body fluids, and highly contaminated waste. HCWs must be trained in infection prevention and control practices and use of PPEs to handle such situations (54, 55).

The knowledge and awareness of the disease are important parameters for the adoption of protective measures that minimize the exposure risk of the illness. Our findings suggest that residents who are less educated and who are non-healthcare professionals possess less knowledge of COVID-19 disease and preventive measures than their counterparts. Therefore, health promotion and awareness programs are warranted to address these particular sections of the population. Thus, COVID-19 awareness programs and other educating strategies should be developed and implemented more effectively to eradicate this disease and increase the breadth of knowledge of rurally and minimally educated populations. These findings are useful for public health policymakers and health workers to recognize target populations for COVID-19 prevention and health education.

The strength of the study lies in its large sample size, recruited during a crucial period—the early stage of the COVID-19 outbreak in Saudi Arabia. Nevertheless, this was an online self-reported survey conducted during lockdown due to pandemic, and this affected our outreach to the general population. Our sample was obviously over-representative of well-educated people, including healthcare workers, and those who have access to computers and the internet. Hence it may not truly represent the entire population of the study region. Therefore, the generalization of the findings may suffer from reporting bias.

## Conclusion

The present study sheds light on the current level of awareness regarding COVID 19, including knowledge, preventative practices, and preparedness in the South-West region of Saudi Arabia, which is still struggling to achieve its target of total COVID-19 eradication. The results of this survey indicated that the majority of respondents were aware of the knowledge, preventive measures and well prepared to fight against COVID-19. It was evident that the community's overall COVID-19 awareness and their preparedness among educated and HCWs populations were fairly satisfactory. However, there were few misconceptions regarding the mode of COVID-19 transmission among the participants, which need to be addressed. Knowledge and preparedness do translate into improved practices toward COVID-19 prevention and the same was reflected in this study. In order to achieve complete control over COVID-19, it would also be worthwhile to invest in various COVID-19 prevention efforts, including health education and innovative strategies based on local evidences to raise the community's awareness and to improve its preventative practices.

## Data Availability Statement

The raw data supporting the conclusions of this article will be made available by the authors, without undue reservation.

## Author Contributions

RT and PT: conceptualization, methodology, writing of the original draft, investigation, project administration, and final editing. SSA and AA: supervision, co-project administration, data collection, feedback, and making substantive changes. AM: software, validation, and formal analysis. SA: visualization and investigation. WA and DB: data collection and calculations, writing, reviewing, editing of the manuscript, and formal analysis. FA: preparation of Google form and Arabic translation. All authors participated in the distribution of the survey.

## Conflict of Interest

The authors declare that the research was conducted in the absence of any commercial or financial relationships that could be construed as a potential conflict of interest.
